# Identify the Alteration of Balance Control and Risk of Falling in Stroke Survivors During Obstacle Crossing Based on Kinematic Analysis

**DOI:** 10.3389/fneur.2019.00813

**Published:** 2019-07-30

**Authors:** Na Chen, Xiang Xiao, Huijing Hu, Ying Chen, Rong Song, Le Li

**Affiliations:** ^1^Department of Rehabilitation Medicine, First Affiliated Hospital, Sun Yat-sen University, Guangzhou, China; ^2^Department of Rehabilitation Medicine, Luo Hu Peoples' Hospital, Shenzhen, China; ^3^Guangdong Work Injury Rehabilitation Center, Guangzhou, China; ^4^Key Laboratory of Sensing Technology and Biomedical Instrument of Guang Dong Province School of Engineering, Sun Yat-sen University, Guangzhou, China

**Keywords:** stroke, gait, balance control, obstacle crossing, kinematics

## Abstract

This study aims to compare the differences in the kinematic characteristics of crossing obstacles of different heights between stroke survivors and age-matched healthy controls and to identify the changes of balance control strategy and risk of falling. Twelve stroke survivors and twelve aged-matched healthy controls were recruited. A three-dimensional motion analysis system and two force plates were used to measure the kinematic and kinetic data during crossing obstacles with heights of 10, 20, and 30% leg length. The results showed that during leading and trailing limb clearance, (AP) center of mass (COM) velocities of the stroke group were smaller than those of the healthy controls for all heights. The decreased distances between COM and center of pressure (COP) in the AP direction during the both trailing and leading limb support period were also found between stroke survivors and healthy controls for all heights. The COM velocity and COM-COP distance significantly correlated with the lower limb muscle strength. In addition, stroke survivors showed greater lateral pelvic tilt, greater hip abduction, and larger peak velocity in the medio-lateral (ML) direction. There was a positive correlation between the COM-COP distance in the AP direction and the clinical scales. These results might identify that the stroke survivors used a conservative strategy to negotiate the obstacles and control balance due to a lack of muscle strength. However, the abnormal patterns during obstacle crossing might increase the risk of falling. The findings could be used to design specific rehabilitation training programs to enhance body stability, reduce energy cost, and improve motion efficiency.

## Introduction

The impairments from stroke impact patients' activities in daily life. Most community-dwelling stroke survivors can walk safely on level surfaces, but they have difficulties in maintaining balance during complex motor tasks such as obstacle crossing (1). Compared with healthy controls, stroke survivors are more likely to fall during obstacle avoidance, either by contacting the obstacle or losing balance (2). The consequences of falling include hip fractures, soft tissue injuries, fear of falling, hospitalization, increased immobility, and greater disability (3, 4). Moreover, Said and colleagues found stroke survivors who failed in the obstacle crossing task demonstrated higher falling risk compared with who passed the task (5). Therefore, identifying falling risk during obstacle crossing and preventing falls are important for stroke survivors (6).

Successful obstacle crossing requires sufficient toe-obstacle clearance provided by the swing limb and body stability provided by the stance limb. This calls for complicated and coordinated controls of both limbs during crossing (7). Researches have been conducted to investigate the motor control strategies among young and old healthy adults and stroke survivors, and find the different strategies could be caused by age-related physical degradation and the stroke-induced muscle weakness (6–9). However, the motor control changes for stroke survivors to make safely step across the obstacle with insufficient muscle strength are still not clear. And quantitative evidence of the balance control changes during such complex task of obstacle crossing for stroke survivors need further investigation. In the previous studies, the most-used strategy that stroke survivors took named circumduction was found during level walking (9), and also the reduced muscle response in stroke survivors compared with healthy controls during obstacle (10). In addition, Lu and colleagues investigated motor performance in highly functional post-stroke patients during obstacle crossing, and found that stroke survivors appeared to adopt a specific symmetric kinematic strategy with an increased pelvic posterior tilt and swing hip abduction (11). Studies included motor control strategies and balance assessment among stroke survivors during such complex task of obstacle crossing are needed.

Balance is often quantified using laboratory-measured variables such as the velocity of the center of mass (COM) and the distance between COM and the center of pressure (COP) (12–15). Also, the Berg Balance Scale (BBS) and the Fugl-Meyer Assessment (FMA) are two clinical measures of balance and motor impairment that are widely used in the field of stroke rehabilitation (16, 17). However, the clinical scales could provide simple assessments and have doubtful ability to demonstrate equivalent quality to laboratory-measured characteristics. Corriveau and coworkers found significant negative linear correlation between clinical scales (BBS and FMA) and COM-COP distance during quiet stance in stroke survivors. They found that postural stability measured by the COM-COP distance was related to the functional measures of balance (18). However, there is little information about the correlation between clinical scales and variables reflecting dynamic balance such as the COM-COP distance during obstacle crossing.

This study aimed to identify the falling risk of stroke survivors during obstacle crossing and to investigate the motor control strategies combined with balance modulation to better understand the way that stroke survivors negotiate obstacles of different heights. We hypothesized that stroke survivors might have different balance control and gait pattern during obstacle crossing which may lead to high risk of falling. The correlation of kinematic data with muscle strength and clinical scales could further provide information and evidence to quantify the balance performance during obstacle crossing for stroke survivors. The findings of current study may help to design training protocol and evaluate rehabilitation interventions for motor recovery in stroke survivors and facilitate the improvement of conducting daily task such as obstacle crossing.

## Methods

### Participants

The subjects included 12 stroke survivors and 12 gender-, age-, and height-matched healthy subjects. The basic characteristics of subjects was displayed in Table 1. The inclusion criteria for the stroke patients were (1) stroke with unilateral hemiparesis lesions confirmed by magnetic resonance imaging or computed tomography; (2) at least 3 months having passed since the stroke; (3) capability of walking 10 meters without a gait aid or assistance and across an obstacle with a height of 30% leg length. The exclusion criteria were other neurologic diseases, such as Parkinson's disease, diabetic polyneuropathy, Alzheimer's disease, and other cognitive impairments. This study was approved by the Ethics Committee of the local hospital and was conducted in accordance to the Declaration of Helsinki. The consent obtained from the participants was both informed and written before the experiments.

**Table 1:** Basic characteristics of study subjects.

**Characteristic**	**Stroke group (*n* = 12)**	**Control group (*n* = 12)**	***p*-value**
Age, years (mean ± SD)	57.4 ± 10.8	59.3 ± 7.1	0.316
Height, cm	165.3 ± 6.6	163.3 ± 5.8	0.156
Mass, kg	64.8 ± 8.5	60.9 ± 7.8	0.262
Brain lesion side, right:left	7:5		
Latency since stroke, months (range)	17.5 ± 17.1 (3–59)		
Berg test scores (range)	43.3 ± 6.7 (27–50)	56 ± 0 (56–56)	<0.001^a^
FMA scores (range)	24.8 ± 3.7 (19–28)	34 ± 0 (34–34)	<0.001^a^
Lower limb muscle strength (mean ± SD), (N)			
Knee extensors	182.7 ± 58.4	263.1 ± 66.4	0.005^a^
Knee flexors	115.8 ± 56.6	192.8 ±43.0	0.001^a^
Ankle dorsiflexors	84.3 ± 42.2	158.6 ± 30.4	<0.001^a^
Ankle plantarflexors	88.4 ± 48.4	175.2 ± 42.5	<0.001^a^

a *Indicates significant effect using an independent t-test. FMA, Fugl-meyer assessment*.

### Apparatus

Thirty-five 15-mm infrared-reflective markers were taped to the skin overlying body landmarks according to the Vicon Plug-In Gait marker placement method. A 6-camera 3D motion analysis system (Vicon Motion Systems, Oxford, UK) recorded the marker positions at a sample frequency of 100 Hz. Two force plates (464 mm × 508 mm × 83 mm; AMTI, Watertown, MA, USA) with a sample frequency of 1 kHz were placed in the middle of the path with obstacles between them. The data from the three-dimensional motion system and the force plates were synchronized. The obstacle has adjustable height and consists of two upright stands with a light-weight crossbar, which was set to three height conditions (10, 20, and 30% of the leg length). A handheld muscle-testing dynamometer (microFET3, Hogan Health, USA with the precision of 0.4 N and range from 13 to 1,330 N) was used to measure the peak isometric force (19).

### Procedure

#### Anthropometric Measure

Before the gait analysis, the basic characteristics were first measured. Leg length was measured with a tape measure from the anterior superior iliac spine to the lateral malleolus and was used to calculate the obstacle height for each individual.

#### Muscle Strength and Clinical Scales

The peak isometric forces of the knee extensors and flexors and the ankle dorisflexors and plantarflexors were also measured. Muscle strength was measured as the peak isometric force in 4 muscle groups including rectus femoris (RF), biceps femoris (BF), tibialis anterior (TA), and medial gastrocnemius (MG) of the affected lower limb of the stroke survivors and the dominant lower limb of the healthy controls (20). Details of the procedures of testing muscle strength could be found in our previous paper (21). An experienced physiotherapist who was blinded to the gait results evaluated the lower extremity FMA and BBS to assess the lower limb motor function and balance function of the stroke group.

#### Kinematic Data

Subjects were then instructed to walk at a self-selected speed with bare feet along an 8-meter walkway, where an obstacle was placed midway, perpendicular to the walking direction, and parallel to the ground. Each trial began at a similar starting point of the walkway with a marker on the floor and the subjects were suggested to use it as a reference of similar walking distance. The stroke survivors were instructed to use their affected leg as the leading limb of the obstacle crossing, and the healthy controls were not restricted of which leg be used. First, the level unobstructed walking was performed, then the different heights were crossed in random order, and three successful trials were recorded for each height condition. We ignored the trials in which the subjects touched the obstacle. Trials were excluded from motion analysis if the participant required the therapist's assistance to maintain balance or tripped over the obstacle. Subjects were reminded to perform the task within their limits of safety and stop if they felt at risk. A therapist accompanied the subjects and walked alongside them to provide assistance if required.

### Data Processing

Vicon Nexus (Version 1.7.1) was used for data processing. The kinematic data were obtained during the crossing stride, which was defined as the period beginning with the trailing limb's heel-contact just before crossing the obstacle to the next heel-contact just after crossing the obstacle (22). The crossing stride can be further divided into five sub-phases: the pre-obstacle double support phase, single support phase of the trailing limb support period (TLP), obstacle-crossing double support phase, single support phase of the leading limb support period (LLP), and post-obstacle double support phase.

**Figure 1:**
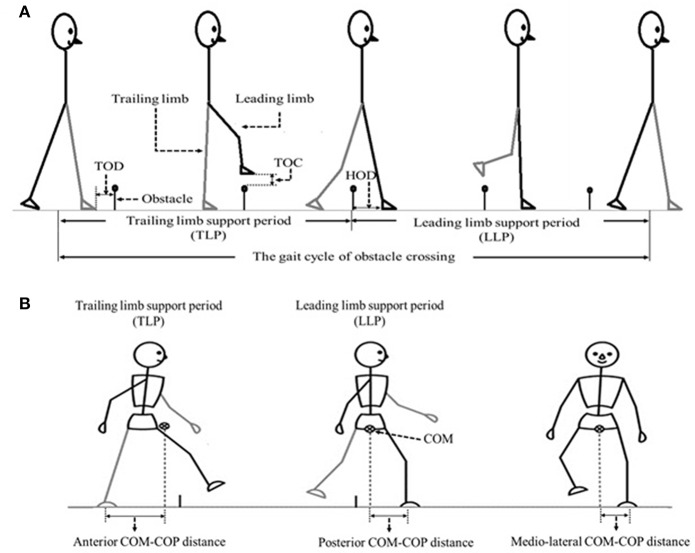
**(A)** The sketch map of the gait cycle of obstacle crossing, including Trailing limb supporting period (TLP) and leading limb supporting period (LLP). TOD (the horizontal distance between the trailing toe (trailing limb is in contact with the floor) and the obstacle before crossing the obstacle), TOC (the vertical distance between the toe of leading toe and obstacle when the toe is over the obstacle) as well as HOD (the horizontal distance between the leading heel (leading limb is in contact with the floor at this time) and the obstacle after crossing the obstacle) were also demonstrated. **(B)** The sketch map of the COM-COP distance at anterior–posterior (AP) and medio-lateral (ML) direction.

The angles of pelvis and lower limb joints of both the stance and swing limbs were calculated when the toe marker was above the obstacle (8). To calculate the end point data (the distance between the toe marker and the obstacle), we defined three distances between the lower limb and the obstacle (Figure 1): TOD is the horizontal distance between the trailing toe and the obstacle before crossing the obstacle; HOD is the horizontal distance between the leading heel and the obstacle after crossing the obstacle; TOC is the vertical distance between the toe of leading toe and obstacle when the toe is over the obstacle. COM and COP locations were calculated using Vicon Workstation software. In details, COM is based on Plug-in-gait model of VICON system with the weighted average value of head, sternum, humerus, radius, hand, pelvis, femur, tibia and foot. COP is based on data from force plates. Instantaneous anterior–posterior (AP) COM velocity was examined at two critical phases: leading limb clearance (LC) and trailing limb clearance (TC) (Figure 1). Medio-lateral (ML) COM velocity was quantified by examining the peak ML velocity during the double support periods: the pre-obstacle (peak 1), during-obstacle (peak 2), and post-obstacle crossing phases (peak 3). The distance between COM and COP was calculated as the root mean square (RMS) during TLP and LLP (18). When examining the correlations between the balance variables and the measured muscle strength, variables representative of obstacle crossing were first averaged within each of the three heights.

### Statistical Analysis

All statistical analyses were performed using IBM SPSS Statistics version 20.0. All the calculated variables for both groups were firstly subjected to a Kolmogorov–Smirnov test. The pelvis and joint angles did not show a normal distribution, and they were represented by the median value with interquartile range (IQR) (1, 5). We then applied the Mann-Whitney U test to these variables and Kruskal-Wallis one-way ANOVA to find the difference of heights within each group. Other variables showing a normal distribution were tested using two-way ANOVA with height as the within-group factor and group as the between-group factors. A *post hoc* test with Bonferroni correction was used to examine group differences among different heights. Pearson product-moment correlations were used to examine the relationship between the balance variables and the measured muscle strength, also between the balance variables and clinical scales. The significance level was set at 0.05.

## Results

**Figure 2:**
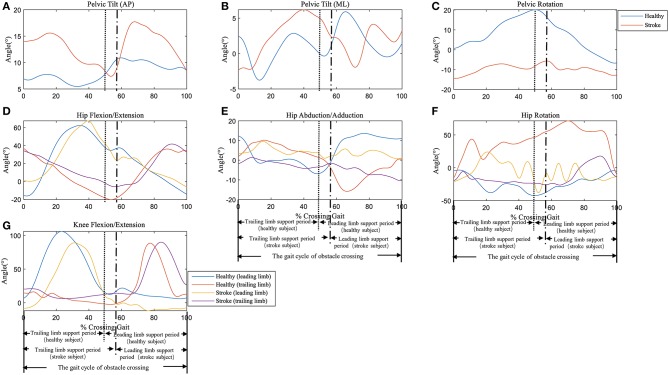
Typical trials of the kinematic behavior of the pelvis at **(A)** Anterior-Posterior (AP) direction, **(B)** Medio-Literal (ML) direction and **(C)** Rotation, and hip joint of **(D)** Flexion/Extension, **(E)** Abduction/Adduction, **(F)** Rotation, as well as **(G)** knee joint Flexion/Extension of the trailing and the leading limb in a patient (female, age 63) and a healthy subject (female, age 62) during the crossing stride sub-phases. See Supplementary Material for all the data trials of stroke and healthy subjects.

Typical trials of the kinematic behavior of the lower limbs and pelvis of the trailing and the leading limb in a patient after stroke and a healthy subject during the crossing stride sub-phases has been shown in Figure 2.

### Kinematic Data of COM-COP

**Figure 3:**
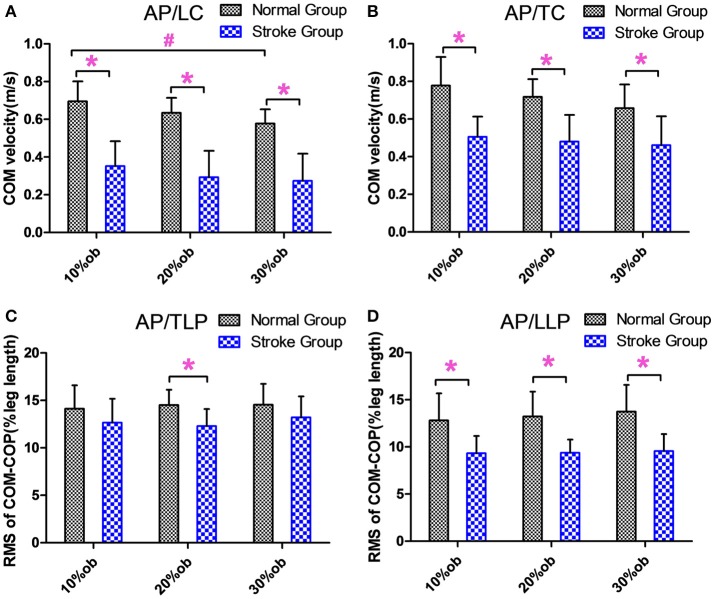
The balance measurements in the AP direction (Mean and SD). **(A)** The velocity at the leading limb clearance. **(B)** The velocity at the trailing limb clearance. **(C)** The COM-COP distance during TLP. **(D)** COM-COP distance during LLP. *Reflects the significant difference between groups, ^#^Reflects the significant difference between heights. The error bar represents 1 SD.

During the leading and trailing limb clearance, the anterior-posterior center of mass (AP COM) velocity of the stroke group was smaller than that of the healthy controls for all obstacle heights (*p* < 0.05, Figures 3A,B), and it decreased with the increasing obstacle height at leading limb clearance (*p* < 0.05, Figure 3A). The AP COM-COP distance of the stroke group during trail limb support period (TLP) showed a significant decrease compared with the healthy controls for 20% height (*p* < 0.05, Figure 3C). During leading limb support period (LLP), the AP COM-COP distance of the stroke group was significantly smaller than that of the healthy controls for all obstacle heights (*p* < 0.05, Figure 3D).

**Figure 4:**
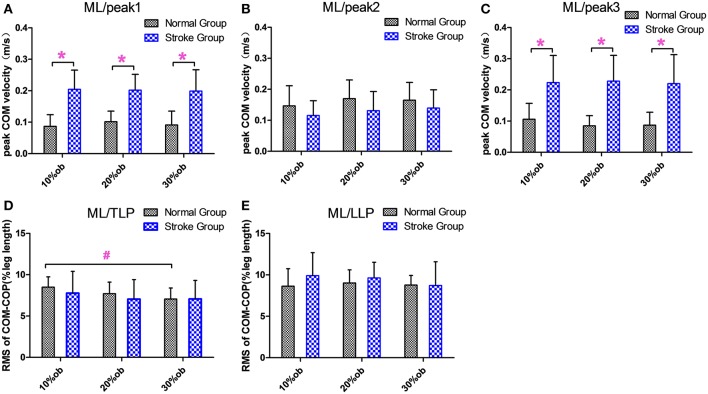
The balance measurements in the ML direction (Mean and SD). **(A)** The velocity at the peak 1. **(B)** The velocity at the peak 2. **(C)** The velocity at the peak 3. **(D)** COM-COP distance during TLP. **(E)** COM-COP distance during LLP. *Reflects the significant difference between groups, ^#^reflects the significant difference between heights. The error bar represents 1 SD.

The peak medio-lateral (ML) COM velocity of the stroke group during the double support phases in both the pre-obstacle (peak 1) and post-obstacle (peak 3) phases were significantly greater than for the healthy controls at all heights (*p* < 0.05, Figures 4A,C). However, there were no significant differences between groups in the peak ML COM velocity during the double support phase (peak 2, *p* > 0.05, Figure 4B). During the TLP, the increasing obstacle heights resulted in decreases in the ML COM-COP distance in both groups (*p* < 0.05, Figure 4D). However, there were no significant differences between groups in the COM-COP distance in the ML direction during TLP and LLP (Figures 4D,E).

### Joint Angle and End Point Data During the Crossing

The crossing pelvis and joint angles data are shown in Table 2. Greater lateral pelvic tilt was found in the stroke group (*p* < 0.05) for all heights compared with the healthy controls. No significant difference was found in the anterior or posterior pelvic tilt or the pelvic rotation. In the leading swing limb, greater hip abduction (*p* < 0.05) and smaller knee extension (*p* < 0.05) were found in stroke survivors compared with healthy controls for all heights. In the trailing stance limb, greater hip abduction and knee extension were also observed in the stroke group compared with healthy controls for all heights (*p* < 0.05).

**Table 2:** Median crossing pelvis and joint angles of the limbs when the leading toe was above the obstacle (Mean (SD), *n* = 12).

**Crossing angle(°)**	**Control**	**Stroke**	***p*-value(group)**	***p*****-value(height)**	
				**Control**	**Stroke**
Pelvic tilt (AP)				0.370	0.881
10%	6.24 (15.39)	6.64 (10.56)	0.843		
20%	3.67 (16.17)	4.83 (7.05)	0.755		
30%	2.31 (13.50)	5.53 (11.16)	0.242		
Pelvic tilt (ML)				P_10%−30%_ = 0.011^b^	0.07
10%	4.50 (3.15)	8.16 (7.19)	0.012^a^		
20%	8.14 (9.38)	12.12 (5.87)	0.048^a^		
30%	11.23 (6.06)	14.75 (5.30)	0.042^a^		
Pelvic Rotation				0.991	0.825
10%	1.13 (6.73)	−6.75 (21.36)	0.219		
20%	1.72 (12.36)	−8.16 (21.38)	0.160		
30%	0.57 (7.17)	−10.07 (22.03)	0.101		
Hip Abduction/Adduction				0.053	0.067
Trailing Limb					
10%	3.68 (4.80)	−3.39 (4.13)	0.001^a^		
20%	0.12 (6.88)	−6.37 (6.45)	0.02^a^		
30%	−3.26 (7.34)	−9.79 (7.50)	0.024^a^		
Leading Limb				0.218	0.636
10%	0.14 (2.97)	−5.23 (4.53)	0.005^a^		
20%	0.07 (2.08)	−8.40 (8.54)	0.004^a^		
30%	−2.42 (5.46)	−8.03 (9.10)	0.024^a^		
Hip Flexion/Extension				0.945	0.980
Trailing Limb					
10%	1.65 (12.78)	7.34 (15.79)	0.242		
20%	4.00 (13.58)	8.21 (16.42)	0.319		
30%	2.26 (7.89)	8.25 (15.85)	0.178		
Leading Limb				0.171	P_10%−30%_ = 0.032^b^
10%	65.51 (17.54)	60.27 (12.18)	0.378		
20%	69.20 (16.20)	70.28 (8.90)	0.887		
30%	75.46 (18.63)	73.19 (14.88)	0.671		
Hip Rotation				0.777	0.831
Trailing Limb					
10%	−2.38 (23.56)	−13.03 (17.90)	0.378		
20%	−9.75 (21.30)	−17.38 (18.68)	0.319		
30%	−9.65 (19.36)	−13.97 (18.51)	0.514		
Leading Limb				0.995	0.846
10%	5.26 (27.41)	2.78 (26.64)	0.713		
20%	−0.67 (37.22)	9.32 (26.06)	0.347		
30%	1.33 (28.99)	9.92 (22.52)	0.347		
Knee Flexion/Extension				0.739	0.889
Trailing Limb					
10%	4.14 (9.55)	13.60 (7.77)	0.004^a^		
20%	8.03 (10.25)	13.48 (6.99)	0.01^a^		
30%	6.56 (11.16)	12.44 (8.40)	0.005^a^		
Leading Limb				P_10%−30%_ <0.001^b^	P_10%−30%_ = 0.0016^b^
10%	86.60 (11.19)	72.88 (31.72)	0.038^a^		
20%	97.46 (15.77)	93.01 (20.86)	0.041^a^		
30%	105.64 (12.11)	96.63 (21.44)	0.046^a^		

a Indicates significant effect between groups.

b Indicates significant effect between heights.

*Pelvic Rotation (anterior +, posterior −); Abduction (+)/Adduction (−); Flexion (+)/Extension (−)*.

Table 3 presents the end point data. Comparisons between the stroke and healthy elderly groups revealed that the stroke group had less HOD (10%: *p* = 0.024; 20%: *p* = 0.01; 30%: *p* = 0.045). No significant differences were found in the leading TOC and the trailing TOD (*p* > 0.05).

**Table 3:** Mean distance (standard deviation) between the limb and the obstacle.

**End point-obstacle distance**	**Control**	**Stroke**	***p*-value** **(group)**	***p*****-value (height)**	
				**Control**	**Stroke**
TOD (%Leg Length)				0.710	0.628
10%	23.24 (5.72)	25.25 (8.80)	0.799		
20%	25.54 (6.01)	22.86 (3.12)	0.101		
30%	26.91 (8.42)	24.25 (5.75)	0.114		
HOD (%Leg Length)				0.662	0.934
10%	17.07 (9.46)	12.78 (7.37)	0.024^a^		
20%	20.18 (6.95)	12.70 (7.89)	0.010^a^		
30%	20.30 (5.14)	13.37 (8.93)	0.045^a^		
TOC (cm)				0.919	0.613
10%	18.72 (5.60)	19.17 (5.96)	0.843		
20%	17.38 (1.79)	18.07 (2.28)	0.319		
30%	16.71 (3.89)	17.10 (4.12)	0.932		

a Indicates significant effect between groups.

*TOD, the horizontal distance between the trailing toe and the obstacle before crossing the obstacle; HOD, the horizontal distance between the leading heel and the obstacle after crossing the obstacle; TOC, the vertical distance between the toe of leading toe and obstacle when the toe is over the obstacle*.

### Correlations Between Kinematic Data, Muscle Strength and Clinical Scales

Table 4 shows the correlation between COM velocity, COM-COP distance and lower limb muscle strength for stroke survivors. The ankle dorsiflexor strength correlated significantly positively with COM velocity in the AP direction when during LC (*r* = 0.623, *p* < 0.05) and TC (*r* = 0.690, *p* < 0.05). The ankle plantarflexors strength correlated significantly positively with COM velocity in the AP direction when during LC (*r* = 0.623, *p* < 0.05). While in the ML direction, there were significant negative correlations between knee extensors strength (*r* = −0.608, *p* < 0.05) and COM velocity during peak 2, between ankle dorsiflexor (*r* = −0.787, *p* < 0.01) and plantarflexors strength (*r* = −0.578, *p* < 0.05) and COM velocity during peak 3. As to COM-COP distance, the ankle dorsiflexor strength correlated significantly positively with it in the AP direction during TLP (*r* = 0.631, *p* < 0.05) and LLP (*r* = 0.694, *p* < 0.05). While the ankle plantarflexor strength correlated significantly positively with it in the AP direction during LLP (*r* = 0.674, *p* < 0.05).

**Table 4:** Correlations between COM velocity, COM-COP distance and lower limb muscle strength.

	**Knee extensors**		**Knee flexors**		**Ankle dorsiflexors**		**Ankle plantarflexors**	
	***r***	***P*-value**	***r***	***P*-value**	***r***	***P*-value**	***r***	***P*-value**
**COM velocity**								
APLC	−0.117	0.718	0.038	0.906	0.623^*^	0.030	0.690^*^	0.013
APTC	−0.135	0.677	−0.265	0.406	0.630^*^	0.028	0.386	0.216
MLP1	0.375	0.230	0.233	0.467	−0.407	0.189	−0.361	0.249
MLP2	–0.608^*^	0.036	−0.398	0.200	−0.395	0.204	−0.354	0.259
MLP3	−0.170	0.598	−0.131	0.684	–0.787^**^	0.002	–0.578^*^	0.049
**Distance between COM and COP**								
APTLP	−0.191	0.552	0.176	0.583	0.631^*^	0.028	0.373	0.233
APLLP	−0.161	0.618	−0.080	0.805	0.694^*^	0.012	0.674^*^	0.016
MLTLP	−0.064	0.843	−0.402	0.195	−0.222	0.488	−0.168	0.601
MLLLP	−0.104	0.748	−0.058	0.858	−0.322	0.307	0.057	0.860

* indicates p <0.05.

** indicates p <0.01.

*AP, anterior–posterior; ML, medio-lateral; LC, leading limb clearance; TC, trailing limb clearance; TLP, trailing limb support period; LLP, leading limb support period*.

**Figure 5:**
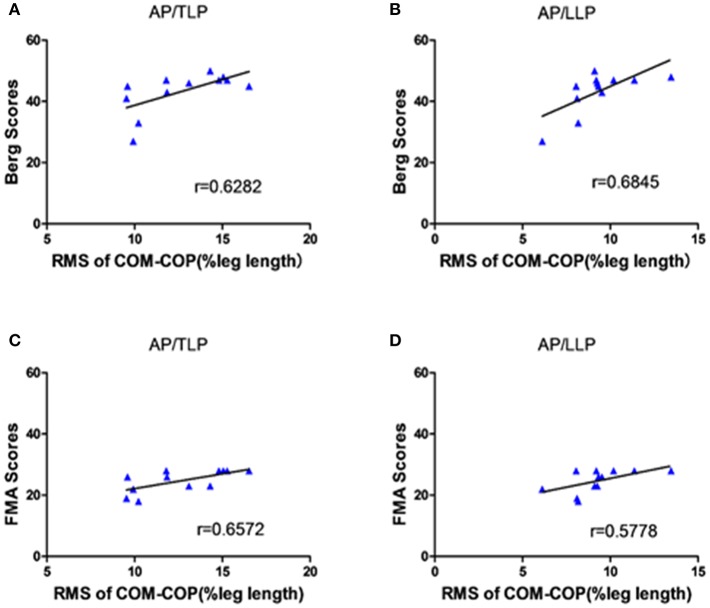
The correlation between the clinical scales and the distance between COM-COP in the AP direction of different periods when crossing the 10% leg length height obstacle. **(A)** The correlation between the BBS scores and the distance of COM-COP during TLP. **(B)** The correlation between the BBS scores and the distance of COM-COP during LLP. **(C)** The correlation between the FMA scores and the distance of COM-COP during TLP. **(D)** The correlation between the FMA scores and the distance of COM-COP during LLP.

Figure 5 shows the significant correlations between the clinical scales and AP COM-COP distance for different periods when crossing the 10% leg length obstacle. The scores of BBS and lower extremity FMA demonstrated moderate positive correlations with AP COM-COP distance (*p* < 0.05). We did not observe any significant correlation between clinical scales and AP COM-COMP at other heights (i.e., 20 and 30%).

## Discussion

We compared kinematic data from stroke survivors and age-matched healthy control subjects when negotiating obstacles of different heights in order to understand the mechanisms of motor control changes during obstacle-crossing after stroke. Our results demonstrated that the stroke survivors might change balance control by using a conservative strategy during the obstacle crossing to ensure safe crossing with a lack of muscle strength and to prevent falls.

### COM-COP Distance and COM Velocity

The results showed that the AP COM velocity of the stroke group was significantly slower than that of healthy controls in both the leading limb clearance and trailing limb clearance (*p* < 0.05, Figures 3A,B). With decreased AP COM velocity, the stroke survivors could better control the anterior movement of the COM, which potentially increased the stability and reduced the risk of losing balance. This finding was similar to those of a previous study (15). In the AP direction, the COM-COP distance was significantly smaller in the stroke survivors than healthy controls only at 20% leg length obstacle during TLP, while it was significantly smaller at all heights during LLP (*p* < 0.05, Figures 3C,D). Maintaining the COM closer to the COP could result in smaller moment arms for the body weight of the stance limb and require less muscular effort to maintain balance (12). Said and coworkers demonstrated no differences between groups in AP COM-COP distance during TLP, but they did not investigate the distance after the clearance (15). Interestingly, in current study when stroke survivors were supported by the affected limb after clearance during LLP, it showed that they had smaller AP COM-COP distances. Our results in part supported Said's findings and further demonstrated poor balance ability for stroke survivors after they crossed the obstacle. The reductions in the COM velocity and COM-COP distance were considered as a conservative strategy used by the stroke survivors to deal with the mechanical challenge during obstacle crossing and to increase stability.

Quick weight shifting to the trailing limb and the lateral pelvic tilt to raise the toe resulted in high instantaneous velocity in the ML direction. The higher ML velocities indicated difficulty in maintaining dynamic stability in the frontal plane and could also reflect difficulty in decelerating COM, which is governed by the relative loading and unloading of the two limbs during double support (23). As a result, the ML COM-COP distance following stroke was not decreased as in the AP direction, and there was no significant difference from the healthy controls (Figures 3D,E). This indicates poor balance maintenance in the ML direction and an increased possibility of falling to the side.

### Joint Angle and the Distance Between Lower Limb and Obstacle

To further examine the control strategy of the stroke survivors, we looked for more details about the kinematics using the pelvic and joint angles. Compared with the healthy controls, stroke survivors showed significantly larger lateral pelvic tilt angles in the ML direction and larger hip abduction angles with both trailing and leading limbs (Table 2). This implies that stroke survivors firstly shifted their weight to the unaffected side, raised the pelvis of the affected side, and abducted the hip to elevate the swing toe. According to neurological development theory, proximal control using the pelvis is more efficient than distal control using the hip or knee (24). Using proximal control, the stroke survivors elevated the toe to maintain a safe clearance between the toe and the obstacle and compensated for the decrease of the knee extension. Lu et al. found that stroke survivors with high motor function adopted an increased posterior pelvic tilt strategy during obstacle crossing (10). In our study, the decrease in the AP COM velocity provided enough time for the lateral pelvic tilt strategy where the stroke survivors elevated the toe and cleared the obstacle in a circumduction, and the decreased AP COM-COP distance improved the stability after the clearance. However, as a result of the lateral pelvic tilt strategy, the stroke survivors showed greater peak ML velocity toward the leading and trailing limbs than the healthy controls during peak 1 and peak 3 (Figures 2A,C), which indicated instability in the ML direction during both push-off and landing phase. Moreover, the HOD was significantly smaller in stroke survivors compared with healthy controls (Table 3), and which might place stroke survivors at risk of actual contact or trip of the obstacle (25). These findings implied a high falling risk for the stroke survivors during obstacle crossing.

### Clinical Correlations

We examined the correlations between the muscle strength of the lower limb muscles and the balance variables to further investigate the cause of the poor balance ability among stroke survivors. The significant correlations (Table 4) might provide evidence that deficit in muscle strength could be a cause of the altered strategies among stroke survivors during obstacle crossing. The COM velocity was reduced which may due to the deficit in muscle strength, and poor balance ability resulted in larger COM velocity in the ML direction and increased the falling risk. Stroke survivors with weaker muscle strength had to place the COM closer to the COP to maintain stability. The different locomotor performance caused by muscle strength has been demonstrated (24), which is similar to our findings and supports that the deficit in muscle strength could be related to the changing of balance control. In addition, the abnormal energy cost among stroke survivors might be another reason during obstacle crossing (26).

One interesting phenomenon in our results was that there were significant positive correlations between AP COM-COP distance and clinical scales (both BBS and lower extremity FMA) for the 10% leg length obstacle height (Figure 5). This provided more information about the balance mechanism adopted after stroke. Our findings showed that stroke survivors with higher clinical scores allowed for a relatively greater AP COM-COP distance to ensure safe crossing compared with those with lower clinical scores when crossing relatively low obstacles. Similarly, a significant negative correlation was reported between the BBS and the COM-COP distance following stroke during quiet stance (18). However, there was no significant correlation between the AP COM-COP distance and the clinical scales when crossing higher obstacles crossing (20% and 30% leg height) which might attribute to challenging task caused high variations among patients. Performance was more disturbed for the higher obstacle during obstacle crossing (27). Stroke survivors could not modulate themselves well when facing higher heights and adopted more abnormal patterns to step across the obstacle. Therefore, the measure of AP COM-COP distance could provide a reliable method for assessing dynamic balance for stroke survivors when crossing relatively low obstacles and to clinically assess balance. Similar tasks were performed in patients with traumatic brain injury, and also through COM-COP distance to demonstrate the patients had difficulty maintaining dynamic stability during obstacle crossing (28). Therefore, the obstacle crossing task challenges stroke survivor's ability to maintain balance, and makes them adopt a quite conservative strategy to safely step across the obstacle. This study can help to understand the control strategy applied by stroke survivors during this complex task and provide understanding to design proper rehabilitation intervention and training theme to decrease the falling risk.

### Limitations

This study has several limitations which need cautions for the interpretation and generalizability of the data. The healthy controls were asked to cross the obstacle with self-selected speed. Previous studies demonstrated that gait changes following stroke were related to speed, and few differences between groups were found when healthy controls used matched speed (25). Moreover, the reductions in the speed of healthy controls might also potentially increase the risk of obstacle contact. Taking these into account, we finally investigated the difference between the two groups at their self-selected speed. In this study, the recruited stroke survivors have moderate to good motor functions and might demonstrated less significant difference compared with healthy controls. We did not record the successful rate of each subject on obstacle crossing but it may be an interesting topic warrant further investigation in the future. In addition, for the safety reason, we did not require the stroke survivor to use the affected side as supporting leg during cross and most of them selected unaffected side to support and all the data been analyzed was from this pattern only. But the healthy subjects were not restricted and they could cross the obstacle using left or right leg and we just found it interesting that most of the heathy subjects using right leg to cross and only those data were analyzed and compared. These might be the reasons that there is no group^*^height interaction effect. In future work, a larger sample size of different kind of stroke survivors as well as an investigation of the mechanism of the neuromuscular activation following stroke [e.g., EMG data (21)] could be added to better examine the biomechanical mechanisms during obstacle crossing.

## Conclusion

The current study investigated the motion patterns in stroke survivors during obstacle crossing compared with healthy controls. The balance of crossing is compromised following stroke and stroke survivors might use a conservative strategy to negotiate the obstacles to prevent tripping which might due to a lack of muscle strength. In addition, there were some abnormal patterns during the crossing, which might increase the risk of falls and instability of balance. The positive correlation between the COM-COP distance and the clinical scales indicates potential as a suitable method for assessing the ability to maintain balance during obstacle crossing. Muscle strength training is recommended for rehabilitation to regain balance ability and to correct the abnormal gait patterns for stroke survivors.

## Ethics Statement

This study was approved by the Ethics Committee of the First Affiliated Hospital, Sun Yat-sen University, and all subjects provided informed consent before the experiments.

## Author Contributions

NC, XX, HH, and LL conceived and designed the study. NC, XX, HH, and YC performed the experiments. NC, XX, HH, and YC wrote the paper. NC helped to response the comments from the reviewers and revise the manuscript. RS and LL made a contribution to experiments. RS and LL reviewed and edited the manuscript. All authors had read and approved the manuscript.

### Conflict of Interest Statement

The authors declare that the research was conducted in the absence of any commercial or financial relationships that could be construed as a potential conflict of interest.
