# Supplementary material for: Identify the Alteration of Balance Control and Risk of Falling in Stroke Survivors During Obstacle Crossing Based on Kinematic Analysis
Source: Front Neurol. 2019 Jul 30;10:813. doi: 10.3389/fneur.2019.00813 (PMC6682676; doi:10.3389/fneur.2019.00813)
Supplement: Supplementary file 1 [file Table_1.docx]

Appendix 1

Table 1 Spatial-temporal parameters of two groups ($\overline{x}$±S)

|  | Cadence  (steps/min) | Walking Speed  (m/s) | Stride Time  (s) | Foot Off  (%) | Single Support  (s) | Double support  (s) | Stride length  (m) |
| --- | --- | --- | --- | --- | --- | --- | --- |
| Control | 100.87±8.87 | 0.92±0.14 | 1.20±0.11 | 62.40±1.79 | 0.44±0.06 | 0.29±0.06 | 1.09±0.09 |
| Stroke | 82.72±13.36 | 0.53±0.17 | 1.49±0.22 | 62.92±3.43 | 0.47±0.07 | 0.46±0.10 | 0.76±0.16 |
| $P$ | 0.001* | ＜0.001* | 0.001* | 0.645 | 0.330 | ＜0.001* | ＜0.001* |

*$p$＜0.05
